# Step Into My Shoes: Empathy and Shared Emotion in Dementia-Caregiving Contexts

**DOI:** 10.1093/geroni/igaf122.286

**Published:** 2025-12-31

**Authors:** Emily Mroz, Jenna Wells, Carolyn Clevenger

## Abstract

Empathic perspective-taking and shared emotion support meaningful connections between caregivers and people living with dementia (i.e., care recipients). Strong connections, in turn, can support shared decision-making and promote wellbeing for both individuals. Yet, empathy and emotional synergy are often strained by interpersonal and cognitive challenges that are common in dementia contexts. To boost investigation into how empathy and shared emotion can develop, and be nurtured, this symposium includes cutting-edge observational and translational research. First, Dr. Brown will present results demonstrating that care recipients’ poor emotion recognition is associated with caregivers’ increased negative affect during interactions. Dr. Wells will complement this by describing findings that behaviorally-coded shared positive emotion in interactions between dementia caregivers and care recipients is associated with better mental health in caregivers. Next, Dr. Huo will describe a mixed-methods examination of the links between perspective-taking and addressing care recipient needs in spousal care dyads. Dr. Mroz will describe a photo captioning intervention that supports dementia-caregiver perspective-taking. She will showcase approaches caregivers can take when engaging in the intervention maximize improvements in perspective-taking. Dr. Kemp will share results from a pilot trial of an improvisation training program for dementia-caregivers. She will share how improvisation training was found to support caregiver empathy. Our discussant, Dr. Clevenger, will synthesize these talks and describe future directions for promoting empathy and shared emotion in clinical and community settings. These presentations will showcase empathy and shared emotion as vital, teachable skills that can be supported through accessible behavioral interventions in dementia-care contexts and beyond.

